# Effects of the Copenhagen Disease Management Program for Type 2 Diabetes on Healthcare Utilization

**DOI:** 10.1155/jdr/8848819

**Published:** 2025-12-11

**Authors:** M. Bender, C. Glümer, K. Vrangbæk, I. Andersen

**Affiliations:** ^1^ Department of Public Health, University of Copenhagen, Copenhagen, Denmark, ku.dk; ^2^ Copenhagen Centre for Diabetes and Heart diseases, Copenhagen, Denmark

## Abstract

**Aims:**

The aim of providing disease management programs (DMPs) for T2D is to increase the understanding of disease etiology, enhance self‐care management, and promote effective strategies for use of healthcare services. Despite large expenses associated with DMPs within universal welfare states, no current evidence exists on healthcare utilization following participation in DMPs.

**Methods:**

The source population covered all individuals living in Copenhagen Municipality with diagnosed T2D during the period 2016–2021 (*n* = 21,132) and of which 2323 individuals were enrolled in the DMP (i.e., cases). Cases were matched with 6812 nonparticipants (i.e., controls) on sociodemography and disease status and information from nationwide registers. Difference‐in‐difference estimates were conducted to assess the effects of the DMP program on the utilization of healthcare services, general practitioner visits, checkups with podiatrists, and diabetic retinopathy screening. We also studied subgroup effects within socioeconomic groups.

**Results:**

Among individuals enrolled in the DMP, the proportion adhering to the recommendations of one yearly visit *increased* for foot (15%) and eye (10%) examinations, while the average number of GP visits *decreased* (−10%). No significant changes were observed among controls. Substantial gains from enrolling in the DMP were seen in selected subgroups of the population; for example, individuals with a long history of T2D increased participation rates in foot (58%) and eye (38%) examinations.

**Conclusions:**

The results suggest that patients with longstanding T2D and their primary GP have insufficient resources or competencies to promote disease management and underline the value of the DMP in promoting care‐seeking and navigating the healthcare system.

## 1. Introduction

T2D has severe implications for the affected individuals and represents a major financial burden on healthcare systems. A patient with diabetes on average costs the Danish state €14,000 per year, of which approximately €5000 is used for municipal care and rehabilitation [1]. In 2009, the increasing and high prevalence of T2D together with positive evidence on the prospects of delaying or halting the progression through lifestyle modifications, medical management, and early interventions compelled the Danish healthcare authorities to implement new methods of treatment and rehabilitation in the form of disease management programs (DMPs) [2, 3]. Today, all patients with newly diagnosed T2D should as a standard be referred to municipal T2D management.

International evidence from randomized controlled trials suggests small benefits in clinical biomarkers of T2D, blood pressure [4–6], mortality, and costs [7] from DMP for diabetes when compared to usual care. Within universal healthcare systems, where access to treatment is (almost entirely) free and should be equal to all citizens, we are however not familiar with population‐wide effect studies of interdisciplinary diabetes management programs.

Studies among individuals with T2D living in universal welfare states have found large differences in utilization of healthcare services, indicating that equality in formal access to services does not necessarily imply equality in the opportunity to benefit from universal access [8]. In Sortsø et al.’s paper on expenses related to healthcare among T2D patients, they show in detail the complex mechanisms of how social inequality among patients with T2D affects their healthcare utilization. They found, despite equal health expenses to healthcare and pharmaceuticals across socioeconomic groups, that individuals with a high educational level improved the composition of services and received more specialized care [9]. Another study by Tapager et al. showed a lower probability of engaging in preventive care and of following through with recommended T2D management plans among disadvantaged individuals [10]. In a recent study of individuals with T2D, Bender et al. found a higher number of booked T2D management program sessions among individuals with lower education, income, and employment status. Nevertheless, this translated into equal or more participated sessions among individuals with higher SEP [11]. In contrast, women with lower SEP and ethnic minorities both booked and participated in more physical training sessions than their counterparts with high SEP. Even in universal welfare states, individuals from higher socioeconomic positions thus generally show a higher understanding of healthcare information, including how to navigate the system, what services they need, and how to advocate for their care.

The aim of this study was to add to existing studies by measuring the effects of the Copenhagen DMP for T2D on specific healthcare services (GP visits and rate of foot and eye examinations) in individuals enrolled in the program compared with matched controls. Further, we wished to study the effects of the program in subgroups of the population.

## 2. Data and Methods

### 2.1. DMPs for T2D

DMPs are an integrated part of many healthcare systems and consist of formalized guides on how to care, educate, and treat patients with specific chronic diseases [12]. These programs most often also include agreements on responsibilities for areas of treatment and care. In Denmark, diabetes management education and rehabilitation are provided by the 98 municipalities, which must conform to national and regional DMPs, which are structured guidelines with some flexibility in the municipal implementation models [13]. Patients with uncontrolled diabetes or complications are also recommended to receive treatment and rehabilitation in hospital clinics, but the primary education and training of newly diagnosed patients, with few exceptions, takes place in municipal centers. Municipal diabetes care is coordinated with general practitioners who are responsible for diagnosis, diabetes specific checkups, and referral to medical specialists [14]. Referral to the municipal program followed regional clinical guidelines, encouraging standard referral of all newly diagnosed patients. However, referral was ultimately subject to GPs’ discretion and clinical judgment, likely resulting in variation in referrals depending on the individual GP responsible for the patient [13]. The GP is responsible for the planning of a minimum of one yearly T2D checkup which must include blood tests to check kidney function, long‐term blood sugar, and lipid status. Additionally, consultations must include a discussion of the patient’s current disease status and potential adjustments to medical and lifestyle treatment. Patients with T2D will naturally also visit their GP in relation to other diseases, symptoms, and preventive care. Further, yearly foot examination and eye examinations every 1–2 years with specialists are also a central part of the recommendations in the guidelines.

Since 2016, the Copenhagen Municipality Center for Diabetes has offered diabetes specific management to all citizens with diabetes. After an electronic referral from a GP has been issued, the municipal T2D center invites the patient to take part in an introductory conversation. At this conversation, the patient will, in collaboration with a primary health professional, develop a personal disease management plan based on the patient’s needs, motivation, and preferences for activities, as a mix of five activities (dietary education, program consultations, telephone conversations, patient education, and physical training). Several tailored sessions are offered to individuals or groups with special needs or preferences, for instance, gender‐separated activities, evening sessions, and the healthcare staff may (with prior consent by the patient) contact patients if they do not show up at scheduled activities. The aim of providing education in disease management is to increase understanding of disease etiology and to empower the individual in improving self‐care management. Self‐care management covers among other strategies to increase adherence to medical treatments and strategies to comply with recommendations for eye and foot examinations.

### 2.2. Study Population Including Matching of Participants With Controls

The source population includes all individuals living in Copenhagen Municipality with diagnosed T2D. During the period January 2016 to April 2021, 2323 individuals with Type 2 diabetes were referred to the DMP. Statistical analyses are all intention‐to‐treat analyses, meaning that all patients enrolled in the DMP are coded as cases irrespective of their actual degree of participation in the diabetes management program. Likewise, all patients originally referred to the DMP are included in the analysis, regardless of whether they died, relocated, were hospitalized, or entered nursing homes during the follow‐up period. No censoring of data was performed; all available data were analyzed according to the original referral group to preserve the real‐world applicability and the integrity of the assigned groups. Our previous analyses however showed that most of the patients referred to DMP also ended up participating in at least one session [15]. Individuals enrolled in the DMP (i.e., cases) were matched with three unique controls. However, in 6% of the cases, we were only able to identify one or two controls. Matching was based on gender, age group, education, employment status, number of comorbidities, year of T2D diagnosis, and ethnicity (categories of matching variables are shown in Table 1). For both cases and matched controls, we defined the preperiod as the 2 years leading up to the scheduled date of the introductory conversation (from now on referred to as the index date) and, conversely, the postperiod as the 2 years after this date. The index date was defined individually for each patient, allowing the pre‐ and postintervention periods to be anchored relative to this time point. During follow‐up, 51 participants (0.4%) died (45 controls and 6 cases). All participants were retained in the intention‐to‐treat denominator irrespective of death, in accordance with the ICH E9 (R1) guidance that intercurrent events should be handled consistently with the prespecified estimand [16].

**Table 1:** Baseline table of individuals included in disease management program (DMP) and controls.

	**Cases,** **n** = 2323	**Controls,** **n** = 6812	**p** **value (chi** ^**2**^ **)**
	**%**	**%**	
Gender (women)	45	45	0.721
Western origin	62	63	0.617
Age (median, p25–75)	59 (51–57)	59 (51–57)	0.738
Age group			0.991
< 50	22	22	
50–59	31	31	
60–69	33	33	
≥ 70	14	14	
Education			0.995
Short	30	29	
Middle	41	41	
Long	29	29	
Employment			0.956
Employed	34	34	
Currently not employed	30	30	
Pensioned	36	36	
Comorbidity			0.986
0	46	47	
1	32	32	
2+	22	21	
T2D year (median, p25–75)	2011 (2007–2015)	2011 (2007–2015)	0.514

### 2.3. Data

Data on enrollment in the diabetes management program were linked to the outcome variables, foot and eye examinations, and GP visits by means of the unique personal identification number given at birth or immigration. In addition, these data were linked to individual‐level covariates on demography (age, gender, and country of origin), socioeconomic position (education and employment status), and disease (comorbidity and year of T2D diagnosis) to control for confounding and to study the effects of the DMP in subgroups of the population. Finally, we also included information on the year of the index date to account for cohort effects.

#### 2.3.1. Demography and Socioeconomic Position

Data on sex, age, and country of origin (categorized into Danish/Western or non‐Western) were obtained from the Danish Central Population Register [17]. Data on education and employment status were obtained from administrative registers in the year preceding enrollment in the DMP. Individuals’ highest recorded educational attainment was categorized according to ISCED11 [18] into short education (primary and lower secondary education; < 2 years of vocational training), medium education (upper secondary and postsecondary education; 2–4 years of vocational training), and long education (tertiary education; > 4 years of education and academic degree completion). Employment was categorized as employed or currently not employed.

#### 2.3.2. Disease Status

Using data from the Danish National Patient Register (NPR) [19] that included ICD‐10 disease codes, we created 20 major disease categories (see Supporting Information for ICD‐10 codes). Individuals with recorded ICD‐10 codes within the past 10 years were identified as having a somatic morbidity. After excluding T2D from the morbidities, we grouped comorbidities into 0, 1, and 2+ number of comorbidities.

### 2.4. Healthcare Utilization

#### 2.4.1. Podiatrists

In Denmark, specialist foot examinations and treatment sessions are provided by state‐licensed podiatrists. One yearly T2D counseling and/or treatment is subsidized by 50% by the Danish state. All treatments are recorded in the Danish National Health Service Register (NHSR) for Primary Care nationwide databases [20].

#### 2.4.2. Ophthalmologists

Diabetic retinopathy screening and treatment is provided by private medical specialists or as ambulatory care at hospitals, is free of charge, and is registered in the DiaBase.

#### 2.4.3. General Practice

All Danish citizens are connected to one primary care GP clinic. Data covering all services by GPs are included in the NHSR.

## 3. Statistical Analyses

Descriptive statistics included tests of differences in the distribution of covariates between cases and controls. Tests of significance for continuous variables (age and year of T2D diagnosis) were conducted using the Wilcoxon test and chi‐square for categorical variables. By means of linear probability models (LPMs), we estimated the change in utilization of healthcare services (percentage points [%pt] for foot and eye examinations and number of GP consultations) for both cases and controls, before (pre) and after (post) the index date. Models for binary outcomes (foot and eye examinations) were fitted within the generalized estimating equation (GEE) framework, with robust (sandwich) standard errors via a REPEATED statement on the unique subject identifier. Predicted probabilities from these models ranged between 0.29 and 0.51 for foot examinations and between 0.34 and 0.36 for eye examinations, confirming that the models are well behaved and that the resulting estimates of absolute risk differences are valid and interpretable. To assess if changes in utilization in healthcare varied between cases and controls, considering both the within‐pair correlation and the time‐varying effects, we calculated difference‐in‐difference (DiD) estimates. The model thus incorporated individual‐level random effects to account for intraindividual correlation in healthcare utilization over time. All individuals contributed complete data for both the pre‐ and postperiods, ensuring that changes reflect within‐person variation rather than differences in group composition. The LPMs within the DiD framework facilitate straightforward interpretation and have been shown to perform well for binary outcomes in DiD settings [21]. Despite comprehensive matching of cases with controls, we included various potential confounders in the statistical model. In line with methodological standards for DiD analyses, we did not match or adjust for baseline healthcare utilization, as this may introduce bias by conditioning on a potential mediator or outcome‐related variable. Instead, our identification strategy relies on the assumption of parallel trends in the absence of referral to DMP.

We conducted a series of subgroup analyses, which, in addition to the above‐described statistical model, also included two‐ and three‐way interaction terms between one group, time, and the covariate as *DMP(case, control)*∗*Time(pre, post)*∗*Covariate(cat_1_,….,var_n_)*. Each analysis included relevant confounders, based on our prior knowledge on causal associations; for example, education was adjusted for age, country of origin, and gender but was not adjusted for employment, income, comorbidity, and years of T2D diagnosis.

## 4. Results

Table 1 shows that cases are comparable with controls regarding all seven measures used for matching.

**Figure 1:**
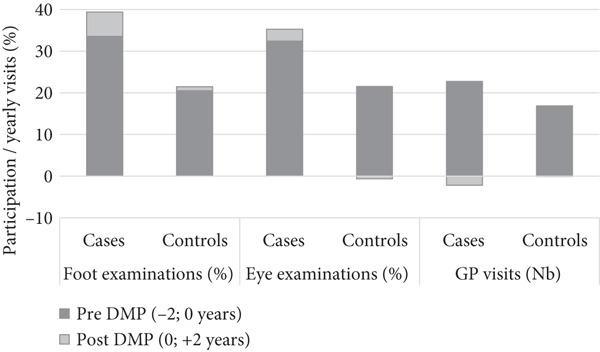
Adjusted rates of healthcare utilization in cases enrolled in disease management program (DMP) versus controls. Supporting data can be found in Supporting Information.

Adherence to recommendations for foot and eye examinations was low among both cases and controls (Figure 1 and Supporting Information (available here)), and in the control group, no overall significant changes in healthcare utilization were observed. When compared to controls, cases had considerably higher participation rates, both before and after participating in the DMP. The DiD estimates showed a larger *decrease* in GP visits (−2.2 visits per year; ~−10%) among cases than controls, corresponding to approximately two yearly visits, and in relative terms a 10% decrease in the number of visits. In contrast, there were large *increases* in foot (4.9%p; ~+15%) and eye (3.3%p; ~+10%) attendance rates among cases compared to controls.

We found quite large differences in the effects in utilization of foot and eye examinations between subgroups of the study population (Table 2). The most pronounced effects in eye examinations were seen in men of non‐Western origin (7.0%pt, +25%), in individuals with long education (7.0 pt, +23%), in individuals without comorbidities (6.0%pt, ~+20%), and in individuals having had T2D for 5 or more years (9.2%pt, ~58%). Correspondingly, these same subgroups also had good effects regarding utilization of foot examinations. There seemed to be little or no effect of the DMP on foot and eye examinations among newly diagnosed patients. Overall, the effects on GP visits were smaller.

**Table 2:** Subgroup analyses of healthcare utilization in cases enrolled in disease management program (DMP) versus controls. Measures are percentages.

	**Cases, n = 2332**			**Controls, n = 6812**			**DID**	**p value^b^**
	**Pre**	**Post**	**p value^a^**	**Pre**	**Post**	**p value^a^**		
Foot examinations								
Country of origin (gender)								
Western_men	35.6	38.6	*0.182*	20.8	23.1	*0.041*	0.8	*0.745*
Western_women	32.3	41.4	*0.001*	17.6	17.7	*0.887*	8.9	*0.002*
Non‐Western_men	29.4	37.6	*0.007*	17.8	18.7	*0.554*	7.3	*0.023*
Non‐Western_women	30.6	35.6	*0.094*	21.8	22.0	*0.907*	4.9	*0.151*
Age (years)								
< 55	32.1	39.2	*0.002*	18.6	21.7	*0.005*	4.2	*0.104*
55–64	34.4	39.9	*0.021*	21.6	22.9	*0.260*	4.0	*0.099*
≥ 65	31.2	36.4	*0.031*	18.3	17.0	*0.271*	6.4	*0.012*
Employment status (age < 65)								
Employed	34.8	39.6	*0.067*	20.4	23.9	*0.004*	1.3	*0.626*
Currently not employed	32.0	39.6	*0.000*	19.8	21.0	*0.242*	6.4	*0.006*
Education								
Short	33.7	36.2	*0.300*	19.2	19.6	*0.734*	2.2	*0.426*
Middle	29.6	36.2	*0.037*	20.4	21.0	*0.694*	5.9	*0.088*
Long	32.9	40.7	*< 0.0001*	19.3	21.0	*0.067*	6.2	*0.002*
Comorbidity								
0	30.3	38.5	*< 0.0001*	18.4	20.1	*0.077*	6.5	*0.003*
1	32.7	39.5	*0.005*	19.6	22.1	*0.031*	4.3	*0.098*
≥ 2	36.8	36.8	*1.000*	21.1	18.8	*0.097*	2.3	*0.467*
Years since T2D diagnosis								
< 2	41.6	41.9	*0.916*	23.5	23.3	*0.850*	0.4	*0.856*
2–4	27.5	34.7	*0.005*	15.7	18.4	*0.018*	4.4	*0.107*
≥ 5	26.3	37.4	*< 0.0001*	17.5	18.8	*0.276*	9.9	*< 0.0001*
Eye examinations								
Country of origin (gender)								
Western_men	32.8	35.6	*0.190*	21.1	20.8	*0.738*	3.1	*0.179*
Western_women	29.2	33.9	*0.065*	16.8	16.5	*0.790*	4.9	*0.061*
Non‐Western_men	28.3	34.7	*0.035*	20.6	19.9	*0.662*	7.0	*0.037*
Non‐Western_women	31.9	32.3	*0.867*	25.5	25.0	*0.733*	1.0	*0.752*
Age (years)								
< 55	28.4	31.6	*0.111*	20.4	21.5	*0.266*	2.1	*0.344*
55–64	33.6	35.9	*0.320*	23.9	22.5	*0.206*	3.7	*0.141*
≥ 65	31.7	36.7	*0.025*	18.3	17.1	*0.280*	6.2	*0.012*
Employment status (age < 65)								
Employed	30.6	34.0	*0.139*	23.1	23.5	*0.697*	2.9	*0.240*
Currently not employed	31.5	33.8	*0.254*	21.7	21.2	*0.606*	2.8	*0.205*
Education								
Short	30.7	31.8	*0.618*	18.6	19.5	*0.387*	0.2	*0.925*
Middle	31.1	31.4	*0.932*	24.7	23.7	*0.548*	1.2	*0.709*
Long	31.1	37.0	*0.001*	20.6	19.6	*0.229*	7.0	*< 0.0001*
Comorbidity								
0	30.0	35.3	*0.006*	21.6	20.9	*0.422*	6.0	*0.003*
1	32.1	35.5	*0.141*	20.6	21.3	*0.559*	2.7	*0.288*
≥ 2	33.4	33.9	*0.879*	20.7	18.9	*0.179*	2.2	*0.476*
Years since T2D diagnosis								
< 2	46.3	45.4	*0.715*	28.7	26.4	*0.035*	1.4	*0.578*
2–4	31.2	33.6	*0.338*	18.1	19.5	*0.235*	1.1	*0.703*
≥ 5	15.8	24.9	*< 0.0001*	15.0	15.0	*0.963*	9.2	*< 0.0001*
GP consultations								
Country of origin, gender								
Western_men	21.1	18.8	*< 0.0001*	15.2	15.3	*0.951*	−2.3	*< 0.0001*
Western_women	24.7	22.3	*< 0.0001*	18.9	19.5	*0.047*	−3.0	*< 0.0001*
Non‐Western_men	21.2	18.6	*< 0.0001*	14.2	13.9	*0.292*	−2.3	*< 0.0001*
Non‐Western_women	23.8	22.6	*0.015*	19.1	18.5	*0.034*	−0.5	*0.353*
Age, years								
< 55	21.3	19.0	*< 0.0001*	15.2	14.4	*<0.0001*	−1.5	*< 0.0001*
55–64	22.3	20.3	*< 0.0001*	17.4	17.0	*0.063*	−1.6	*< 0.0001*
≥ 65	24.2	22.0	*< 0.0001*	17.7	18.9	*<0.0001*	−3.4	*< 0.0001*
Employment status (age < 65)								
Employed	20.3	17.7	*< 0.0001*	15.2	14.1	*<0.0001*	−1.4	*< 0.0001*
Currently not employed	23.3	21.5	*< 0.0001*	17.3	17.2	*0.639*	−1.7	*< 0.0001*
Education								
Short	23.1	20.9	*< 0.0001*	16.8	17.4	*0.022*	−2.8	*< 0.0001*
Middle	23.0	21.4	*0.001*	17.6	17.1	*0.105*	−1.1	*0.044*
Long	22.1	19.8	*< 0.0001*	16.3	16.2	*0.307*	−2.1	*< 0.0001*
Comorbidity								
0	22.1	20.4	*< 0.0001*	15.5	15.6	*0.843*	−1.8	*< 0.0001*
1	21.7	19.8	*< 0.0001*	16.8	16.9	*0.571*	−2.0	*< 0.0001*
≥ 2	22.6	19.8	*< 0.0001*	16.8	16.6	*0.450*	−2.6	*< 0.0001*
Years since T2D diagnosis								
< 2	20.9	18.6	*< 0.0001*	17.0	16.5	*0.010*	−1.8	*< 0.0001*
2–4	23.3	20.7	*< 0.0001*	16.7	16.9	*0.385*	−2.8	*< 0.0001*
≥ 5	26.2	24.8	*0.013*	16.8	17.6	*0.019*	−2.1	*0.001*

^a^Type II test for significance between before and after DMP.

^b^Type III test for interaction; significance in change between cases and controls.

## 5. Discussion

Before enrollment in the DMP, cases had considerably higher utilization of healthcare services than controls. While no significant overall change in healthcare service utilization was observed during the study period among controls, cases’ average attendance rates of foot and eye examinations increased following enrollment in the DMP, and the average number of GP visits decreased. We considered clustering analyses at the GP level; however, due to the limited number of cases per GP (range 1–51, p25–p75 1–5), these analyses were not feasible, which would have allowed us to estimate the distinct effects of GP referral behavior and participation in the DMP on healthcare utilization. However, our study still captures the real‐world impact of referral to a municipal diabetes management program by incorporating both the referral decision and subsequent program participation. This reflects how the intervention operates in routine practice, where referral and participation are sequential and interconnected steps along the treatment pathway.

Overall, the DMP had modest to good effects on health service utilization. However, our results suggest that certain groups with T2D may benefit substantially more from participating in a DMP than others. We found the most pronounced effects among men with non‐Western backgrounds, individuals with no comorbidity, and those having had T2D for 5 or more years. Individuals with no prior comorbidity may never have received disease management education, and the program is therefore likely to be particularly beneficial for these individuals. On the other hand, individuals having had T2D for longer periods are more likely to experience complications from their disease, and GPs may feel that they lack the specialized resources, time, or structured approach needed to fully address the complex and ongoing management of T2D.

Individuals younger than pension age showed no significant effects on utilization of foot examinations and overall had much lower effects compared to individuals above the age of 65. Younger individuals might not experience significant symptoms or complications if the disease is in the early stages, which may reduce their motivation to attend regular checkups. They are likely to have better general physical functioning, and a large share will still be active on the labor market. Despite the provision of evening counseling and training at the diabetes center, they may not feel that they have sufficient time or energy to engage in the DMP besides working. This is supported by the results on eye examinations, showing small (and nonsignificant) effects of DMP in individuals with current employment.

Furthermore, the results of this paper suggest that there is a substitution effect, where individuals tend to seek care, guidance, and treatment by eye and foot specialists instead of from their GP [22–24]. We hypothesize that during the DMP, patients are educated to better navigate the healthcare system, directing control, care, and treatment to organ‐specific medical specialists. Indeed, if comparable studies can find support for the substitution effects of DMP, this suggests an optimization of resources as patients are directed towards intended specialized treatment.

### 5.1. Strengths and Limitations

The main strength of this paper is the uniqueness of the data. No previous research has studied the effects of an interdisciplinary DMP for T2D within a universal welfare state. Linking referral data to register data on healthcare service utilization over time has given us the possibility to make causal inferences about the effects of the DMP, as we had complete data on all individuals with diagnosed T2D in Copenhagen Municipality. Data on healthcare utilization has high validity as these data are used for reimbursement of services provided. Further, reflection of real‐life practice is a major strength of intention‐to‐treat analyses, in this study, based on an entire population.

The results of this DMP, developed in Copenhagen Municipality, might not be directly generalizable to other municipalities or countries with different healthcare structures, patient behaviors, or resource availabilities. Nonetheless, the recommendations for T2D management education and training are relatively comparable across nations, and patients with T2D are remarkably equal in terms of disease‐specific challenges and experiences of complications [25, 26].

Despite high concordance between cases and controls in terms of sociodemographic factors, healthcare utilization varied largely before the index date (date of case participation in DMP), suggesting that these groups are less comparable than originally considered. We chose to base our conclusions on DiD analyses, taking differences in baseline healthcare utilization into account. We intentionally did not match or adjust for baseline healthcare utilization, in line with DiD methodology, as conditioning on postbaseline or outcome‐related variables could introduce bias. Patients entered treatment at different times between 2016 and 2021. While more advanced staggered DiD estimators could address such timing variation more precisely [27], our study was limited by one annual measurement per patient and sample size constraints. We acknowledge the methodological value of staggered adoption DiD models and look forward to applying them in future work using larger datasets (e.g., nationwide registers), where such approaches would be more feasible. To account for temporal heterogeneity, we included interaction terms with referral year and conducted stratified robustness checks, which supported our findings. Lastly, it is worth discussing the effect sizes of the results; does an average +15% increase in healthcare utilization represent an effective program? What we can deduce from our results should be judged by health professionals and will need to be supported with analyses of disease progression and use of medication as outcome measures.

## 6. Conclusions and Implications

In this DiD analysis, enrollment in a DMP for T2D was associated with increased compliance with recommendations for yearly attendance of foot and eye examinations and a reduced number of GP visits. We found large variations between subgroups of the population and no effects among individuals with newly diagnosed T2D. The particularly positive effects among patients having had T2D for more than 5 years indicate that these patients and their GPs have insufficient resources or competencies to promote long‐term disease management and underline the value of the DMP in promoting care‐seeking and navigating the healthcare system.

In the current situation, with generally low adherence to recommended guidelines, individuals are unlikely to receive the full benefits of the DMP. The DMP was not successful in improving younger individuals’ healthcare utilization. In the future, efforts should emphasize the importance of regular checkups with specialists for retinopathy and podiatric care to enhance early detection and prevention of complications in this group. These measures are critical in preventing long‐term complications and maintaining overall health. As individuals not referred to DMP have a much lower healthcare utilization, they have more room for improvement if adherence can be increased. Failing to engage a larger share of the T2D patients, and specifically those in most need of support in care‐seeking, means missing an opportunity to improve public health outcomes.

## Ethics Statement

In Denmark, researchers are allowed to use register data for conducting statistical analyses of population‐level outcomes if they comply with a list of predefined ethical standards. Hence, no specific ethical approval was needed for the study.

## Disclosure

The research presented in this paper is original and has not been published or presented previously, nor is it under consideration for publication elsewhere. All authors critically revised the manuscript and approved the final version of the manuscript.

## Conflicts of Interest

The authors declare no conflicts of interest.

## Author Contributions

M.B. is the guarantor of this work and, as such, had full access to all the data in the study and takes responsibility for the accuracy of the analysis. C.G. together with colleagues from the Copenhagen Center for Diabetes was responsible for DMP data collection. M.B. conducted the analysis and was responsible for writing the manuscript. M.B., K.V., and I.A. were involved in the conception and design of the study and discussed data analyses. M.B. is responsible for the integrity of the work as a whole.

## Funding

The study was funded by Novo Nordisk Fonden (10.13039/501100009708), Health Foundation (10.13039/501100000724, 2014B141), Committee for Social Inequality, and Danish Cancer Society Research Center (10.13039/100015459, R73‐A4741‐13‐S17).

## Supporting information


**Supporting Information** Additional supporting information can be found online in the Supporting Information section. Adjusted ^∗^ Difference‐in‐difference (DiD) estimates of healthcare utilization in cases enrolled in disease management program (DMP) versus controls.

## Data Availability

The data that support the findings of this study are available from Statistics Denmark and Copenhagen Center for Diabetes. Restrictions apply to the availability of these data, which were used under license for this study. Data are available from the authors with the permission of Statistics Denmark and Copenhagen Center for Diabetes.
