# Supplementary material for: Effects of the Copenhagen Disease Management Program for Type 2 Diabetes on Healthcare Utilization
Source: J Diabetes Res. 2025 Dec 11;2025:8848819. doi: 10.1155/jdr/8848819 (PMC12767223; doi:10.1155/jdr/8848819)
Supplement: Supplementary file 1 — Supporting Information Additional supporting information can be found online in the Supporting Information section. Adjusted ∗ Difference‐in‐difference (DiD) estimates of healthcare utilization in cases enrolled in disease management program (DMP) versus controls. [file JDR-2025-8848819-s001.pdf]

S1 Online Supplementary Materials. Adjusted* Difference-in-Difference (DiD) estimates of healthcare provision in cases enrolled in disease management program (DMP) vs. controls.

	Cases, n=2332			Controls, n=6812		
	%	CI_low	CI_high	%	CI_low	CI_high
**FOOT examination, participation %**						
Pre DMP (0; - 2 years)	33.6	31.4	35.7	20.5	19.1	21.9
Post DMP (0; +2 years)	39.4	37.2	41.6	21.5	20.1	22.8
P-value (post vs. pre)		*<0.001*			*0.154*	
DiD (%pt [p-value])			4.9 *(0.001)*			
**EYE examination, participation %**						
Pre DMP (0; - 2 years)	32.4	30.3	34.5	21.6	20.2	22.9
Post DMP (0; +2 years)	35.3	33.2	37.4	20.9	19.6	22.2
P-value (post vs. pre)		*0.029*			*0.317*	
DiD (%pt [p-value])			3.3 *(0.014)*			
**GP, mean Nb yearly visits**						
Pre DMP (0; - 2 years)	22.8	22.2	23.3	16.9	16.5	17.2
Post DMP (0; +2 years)	20.6	20.1	21.1	16.9	16.5	17.2
P-value (post vs. pre)		*<0.001*			*0.995*	
DiD (%pt [p-value])			-2.2 *(<0.001)*			

* Adjusted for gender, age, country of origin, education, employment status, comorbidities, yr of T2D and yr of enrollment in the study.
